# Cross-cultural validation of the Dutch Vertigo Symptom Scale – Short Form: dimensionality, reliability and cultural differences in symptom reporting

**DOI:** 10.1186/s41687-026-01109-x

**Published:** 2026-06-10

**Authors:** Anisa S. Y. Noordzij, Berend Terluin, Johannes C. van der Wouden, Henriëtte E. van der Horst, Rosie Essery, Lucy Yardley, Otto R. Maarsingh, Vincent A. van Vugt

**Affiliations:** 1 Department of General Practice, Amsterdam UMC, Vrije Universiteit Amsterdam, Amsterdam Public Health Research Institute, de Boelelaan 1117, Room J2-129, AMC, Amsterdam, WC 1100 Netherlands; 2 Primary Care Research Centre, University of Southampton, Southampton, UK; 3 Department of Psychology, University of Southampton, Southampton, UK; 4 School of Psychological Science, University of Bristol, Bristol, UK

**Keywords:** General practice, Primary care, Dizziness, Cross-cultural validation, Translation vertigo symptom - scale short form

## Abstract

**Background:**

The Vertigo Symptom Scale – Short Form (VSS-SF) is a widely used questionnaire to measure vestibular symptoms such as dizziness and vertigo. Most cross-cultural validations focus only on translation and basic psychometrics, providing limited insight into cross-national comparability. This study aimed to (1) develop and validate a Dutch version of the VSS-SF, and (2) provide novel insights into cross-cultural differences in symptom reporting and the methodological robustness of the scale.

**Methodology:**

Following international guidelines, we translated and linguistically validated the Dutch VSS-SF. We then compared its dimensional structure, reliability, and differential item functioning (DIF) with the original British version, using bifactor modeling to test essential unidimensionality. We used data from patients aged 50 years and older with chronic vestibular symptoms who participated in British (*n* = 296) and Dutch (*n* = 322) randomized controlled trials.

**Results:**

The Dutch version was well understood. Psychometric evaluation supported essential unidimensionality in both versions (structural coefficient bias < 15%). Reliability was excellent (omega-total 0.920 Dutch; 0.956 British). DIF analysis revealed cultural differences: Dutch patients were less likely to report nausea/vomiting but more likely to report unsteadiness, even at the same symptom level. However, these differences had negligible impact on total scores.

**Conclusions:**

The Dutch VSS-SF is valid and reliable, with scores directly comparable to the British version. Beyond translation, this study shows how advanced psychometric methods reveal both the robustness of the VSS-SF and subtle cultural differences in symptom perception, strengthening its use in multinational research and clinical practice.

**Supplementary Information:**

The online version contains supplementary material available at 10.1186/s41687-026-01109-x.

## Background

Vestibular symptoms, such as dizziness and vertigo, are relatively common in general practice consultations, with reported prevalences up to almost 10% [1]. Likewise, in the general adult population, the 12-month prevalence of these symptoms is 15% [2]. As vestibular symptoms cannot be measured objectively, researchers have to rely on patient-reported outcome measures for their quantitative assessment. The Vertigo Symptom Scale – Short Form (VSS-SF), derived from the 27-item Vertigo Symptom Scale developed in the United Kingdom (UK) [3], is a questionnaire that measures the frequency of dizziness, vertigo, imbalance, and related autonomic symptoms (nausea, sweating, etc.) [4]. The VSS-SF consists of 15 items to which patients can respond on a scale from 0 (never) to 4 (very often) to indicate how frequently they experienced vestibular symptoms during the past month (see appendix A for the original British English questionnaire) [5]. The VSS-SF has been translated and cross-culturally validated in several languages such as Japanese, the central Kurdish dialect and Norwegian [6–8]. However, differences in symptom perception and reporting across cultures may affect comparability, underlining the need for rigorous cross-cultural validation beyond translation alone. A validated Dutch version is urgently needed to support its growing use as an outcome measure in clinical trials on vestibular rehabilitation and to enable general practitioners to more accurately assess vestibular symptoms in daily care. Moreover, establishing its dimensional structure and measurement invariance is key to ensure that scores are comparable across countries. The aim of the present study was [1] to develop a cross-culturally adapted Dutch version of the VSS-SF and [2] to provide novel insights into cross-cultural differences in symptom reporting and the methodological robustness of the scale.

The cross-cultural adaptation included a careful British English-to-Dutch translation, followed by a linguistic validation ensuring that the Dutch version is properly understood by the Dutch target population. The psychometric validation entailed a comparison of Dutch and British VSS-SF regarding the scale’s dimensional structure, reliability, and whether the language versions measured the same construct (i.e., vestibular symptoms).

## Materials and methods

### Translation and cross-cultural adaptation

We followed international guidelines for cross-cultural adaptation to develop the Dutch VSS-SF [9]. First, independent forward translation was performed by two Dutch native speakers with adequate British language proficiency and expertise in the field of vestibular symptoms (OM and VV). Together they synthesized a Dutch version of the forward translations, resolving any discrepancies. Secondly, this consensus version was sent to a professional translator at an independent translation agency. This British native speaker, who possessed no specific knowledge of vestibular symptoms or the VSS, used the Dutch translation to create a British back translation. Third, a subset of the research group (OM, VV, JW and HH) compared the original version, the Dutch consensus version and the British back translation with each other, discussing and resolving any discrepancies. This resulted in the provisional Dutch version of the VSS-SF, ready to be tested for comprehensibility using cognitive interviews.

We invited Dutch patients who had participated in a randomized controlled trial that investigated the effectiveness of a multifactorial intervention for chronic dizziness in older people (≥ 65 years) in general practice in 2016 [10]. At the time, chronic dizziness was defined as a giddy or rotational sensation, loss of balance, faint feeling, light-headedness, instability, and/or tendency to fall. None of the patients had ever seen the VSS-SF before. We sent invitation letters to all patients who previously gave permission to be approached to participate in future studies (*n* = 100). We excluded patients who were unable to complete questionnaires and/or partake in interviews due to severe physical or mental illness. The aim was to include between five and eight patients, in accordance with current recommendations [11]. A researcher (AN) interviewed and observed the eligible and consenting patients while they were filling out the Dutch version of the VSS-SF. This was done following the Three-Step Test Interview (TSTI) method in order to assess the comprehensibility of the Dutch version of the VSS-SF [12]. The TSTI procedure consists of [1] observation of response behavior while the participant thinks aloud [2], follow-up probing to remedy gaps, and [3] debriefing aimed at eliciting experiences and opinions. The interviews were audio recorded and transcribed verbatim. A subset of the research team (JW, OM and VV), discussed the transcribed interviews to determine whether the final Dutch version of the VSS-SF needed to be amended.

## Psychometric validation

### Patients

We used baseline VSS-SF scores from two randomized controlled trials that investigated the effectiveness of online vestibular rehabilitation in patients aged 50 years and older with chronic vestibular symptoms [13, 14]. The British patients had filled out the original VSS-SF, whereas the Dutch patients had completed the Dutch version. The trials applied the same eligibility criteria. The inclusion criteria were access to the internet and persisting vestibular symptoms at time of inclusion that had been present for at least one month and were exacerbated or triggered by head movements. With these inclusion criteria participants with a chronic vestibular syndrome, as defined in the International Classification of Vestibular Disorders, were identified [15]. Exclusion criteria were: identifiable non-vestibular cause of their symptoms, medical contraindications for making head movements (e.g., severe cervical arthrosis), serious comorbid conditions precluding participation in an exercise programme, and current enrolment in another similar study.

**Fig. 1:**
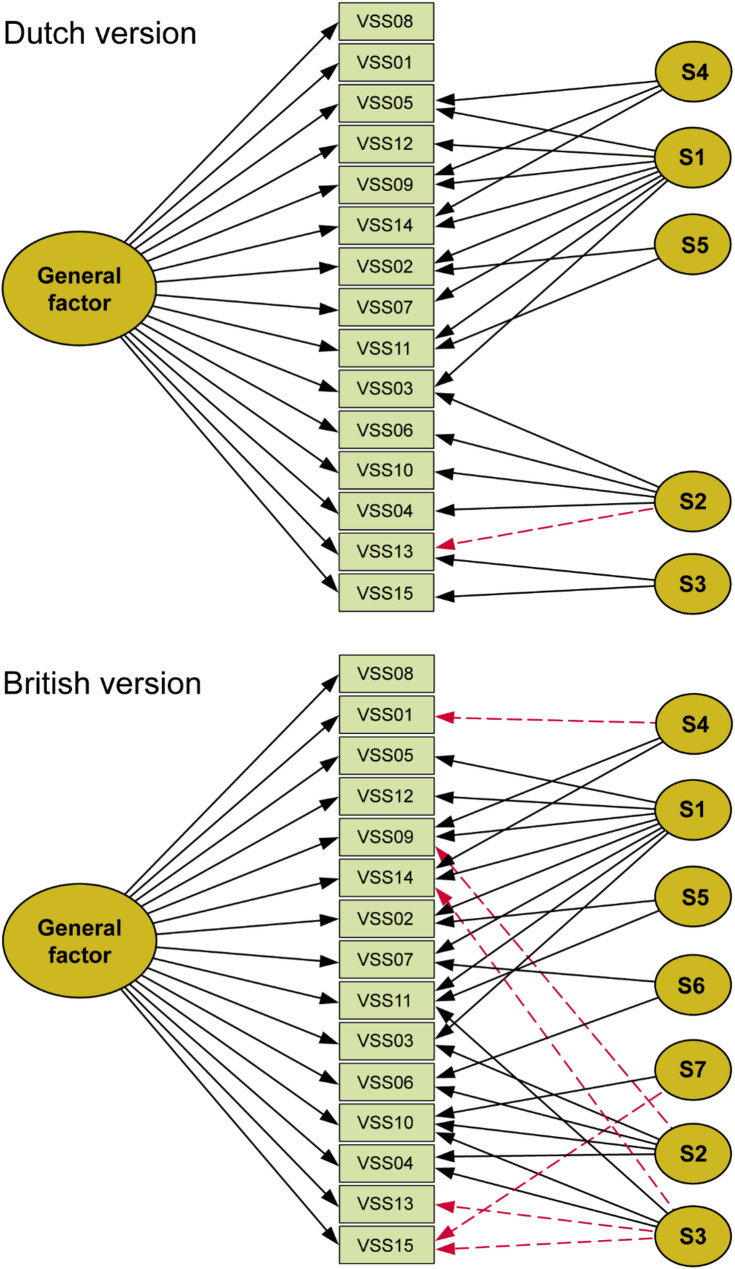
Bifactor structure of the Dutch and British versions of the VSS-SF. Rectangles represent observed item scores. Ovals represent latent variables (factors). Arrows represent factor-item paths (see Table 3 for the standardized factor loadings). Solid arrows represent paths with positive factor loadings, dashed arrows represent paths with negative factor loadings

**Fig. 2:**
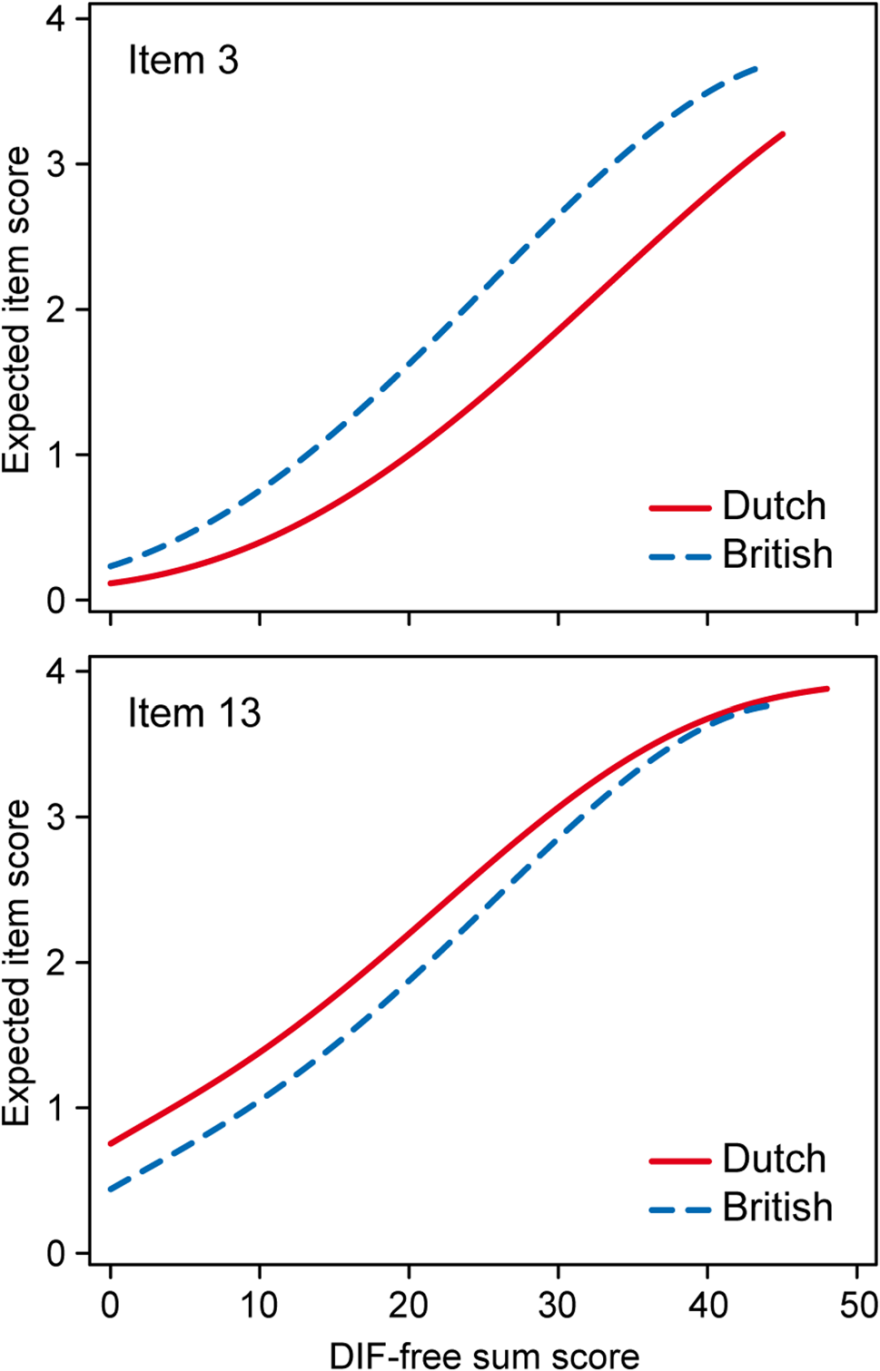
Item-level differential item functioning (DIF). Expected item score as a function of the total score of the DIF-free items, and group (Dutch versus British). Item 3: “Nausea (feeling sick), vomiting”. Item 13: “Feeling unsteady, about to lose balance, lasting less than 20 minutes”

**Fig. 3:**
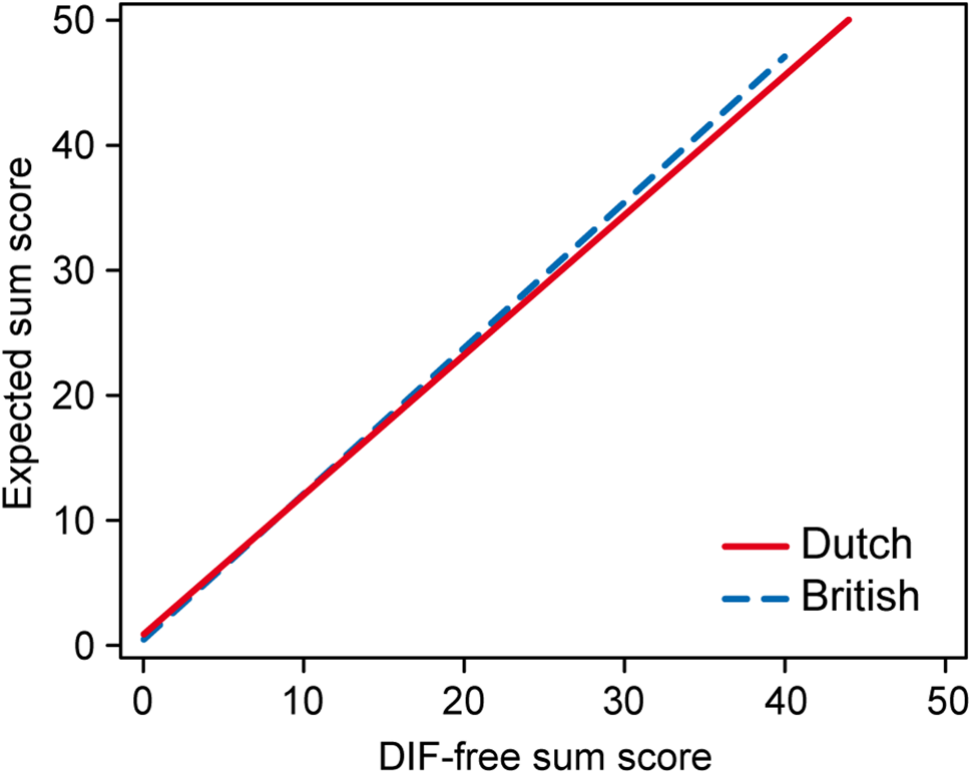
Scale impact of differential item functioning (DIF). Expected sum score (total scale score) as a function of the total score of the DIF-free items, and group (Dutch versus British)

### Dimensionality

The results of an assessment using the VSS-SF is typically expressed as a total (sum) score of all 15 items. Nevertheless, exploratory factor analysis revealed the presence of two or three factors in the Norwegian [8] and Japanese [6] VSS-SF version respectively. The two-factor solution showed an “autonomic-anxiety” factor, with symptoms like excessive sweating and heart pounding, and a vertigo-balance factor, with symptoms like vertigo, unsteadiness and dizziness [8]. The three-factor solution showed practically the same “autonomic-anxiety” factor (only one item different) and two “vestibular-balance” factors, one with symptoms of long duration (> 20 min) and one with symptoms of short duration (< 20 min) [6]. 

There is a risk to treat a multidimensional scale as a unidimensional scale. Respondents with the same (composite) total score might not have the same underlying problem, and the relationship between the construct measured and another (e.g., causal) construct might be underestimated, which threatens the validity of its total scores. However, if the factor structure is dominated by a large “general factor”, the scale might be unidimensional enough to justify the use of total scores. This is called “essential unidimensionality” [16]. We examined if essential unidimensionality was present by fitting a bifactor model to the data using confirmatory factor analysis. A bifactor model is characterized by a large general factor on which all items load, and one or more smaller “specific factors” on which subsets of items load [17]. The factors are orthogonal (i.e., they do not correlate). We allowed items to load on more than one specific factor (so-called unrestricted model). Because the factors in a bifactor model are orthogonal, the model allows for the partitioning of the variance across the general factor, the specific factor(s) and measurement error. We evaluated the model fit by the fit indices and benchmarks proposed by Hu & Bentler: [18] comparative fit index (CFI) > 0.95, Tucker-Lewis index (TLI) > 0.95, root mean squared error of approximation (RMSEA) < 0.06, and standardized root mean squared residual (SRMR) < 0.08. The items were treated as ordered and the estimation used diagonally weighted least squares (DWLS). We started by fitting a one-factor model for comparison, then we fitted a bifactor model with two specific factors resembling the factors in the Wilhelmsen et al. study [8], and a bifactor model with three specific factors resembling the factors in the Kondo et al. study [6]. Then we used the modification indices, in particular the standardized expected parameter change (if > 0.2) to guide model improvement [19]. 

Based on the optimally fitted bifactor model, we estimated omega-hierarchical (omega-h) as a measure of the relative strength of the general factor, and the structural coefficient bias as a direct test of the bias due to treating a multidimensional scale as were it unidimensional.

Omega-h represents the proportion of the sum score variance that is explained by the general factor [20]. We also calculated omega-group (or omega-subscale), which indicates the proportion of subscale score variance that is explained by a specific factor over and beyond the variance that is explained by the general factor [21]. In the context of structural equation modeling, a structural coefficient indexes the strength of the relationship between two latent variables. It can be a correlation or a regression coefficient. If a measurement model defining a latent variable is mis-specified (e.g., if a multidimensional scale is modeled as a unidimensional scale), the latent variable is not well defined and consequently the estimated structural coefficient is biased (typically underestimated). However, if the bias is not too much, the scale can be treated as (essentially) unidimensional. According to Muthén et al., a relative bias of no more than 10–15% may not be serious in most contexts [22]. Structural coefficient bias estimation works as follows: from a well-fitting bifactor model, a matrix of standardized factor loadings is obtained, with items in the rows and factors in the columns [17]. Then, an additional factor (latent variable) is specified with three indicators (items) with standardized factor loadings of 0.60, 0.65 and 0.70, and the additional factor and its indicators are added to the matrix of factor loadings. Then, a matrix of factor intercorrelations is specified, with a correlation of 0.50 between the general factor from the bifactor model and the additional factor. Next, a matrix of inter-item correlations is calculated based on the matrix of factor loadings and the matrix of factor intercorrelations. Finally, the reproduced matrix of correlations is used to fit a correlated two-factor model with one factor on which the questionnaire items are loading and another factor on which the additionally specified items are loading. The correlation between the factors is freely estimated. Note that this procedure forces the multidimensional VSS-SF data into a unidimensional measurement model. The relative extent to which the estimated correlation between the factors deviates from the simulated (“true”) value of 0.5 defines the structural coefficient bias. For comparison, we performed the same analyses in the British data.

### Reliability

As a measure of reliability, we estimated omega-total, representing the proportion of reliable sum score variance in the total sum score variance [20]. For comparison, we also estimated Cronbach’s alpha. Note, however, that Cronbach’s alpha underestimates the true reliability if the scale is not unidimensional and the items do not have the same factor loading on the underlying factor [23]. 

### Differential item functioning

Cross-cultural validity of the Dutch VSS-SF means that the Dutch questionnaire measures the same construct (i.e., vestibular symptoms) in Dutch respondents as the original British questionnaire does in British respondents. It requires that the items of the questionnaire function the same as indicators of the construct across Dutch and British respondents. This so-called measurement equivalence can be tested using differential item functioning (DIF) analysis [24]. For DIF analysis we used ordinal logistic regression (OLR) analysis [25]. Ideally, the items of a questionnaire only measure the construct the questionnaire is purported to measure, whereas other respondent characteristics (e.g., cultural background and language) do not impact the item response. DIF occurs between two groups when item responses, after adjusting for the underlying construct, are affected by group membership. In other words, when respondents from one group achieve higher scores on certain items than respondents from another group, despite having the same level of the construct the item is supposed to measure. There are two types of DIF, uniform and nonuniform. Uniform DIF occurs if one group scores systematically higher on an item than another group, irrespective of the level of the construct. Nonuniform DIF occurs if the degree to which one group scores higher on an item depends on the level of the construct. DIF analysis using OLR examines the contribution of group membership to the prediction of ordinal item scores, while adjusting for the level of the construct. This is done by evaluating three OLR models:

Model 1: $$\:\mathrm{l}\mathrm{n}\left(\frac{P\left(Y\ge\:k|\theta\:,G\right)}{P\left(Y<k|\theta\:,G\right)}\right)={\beta\:0}_{k}+\beta\:1*\theta\:$$

Model 2: $$\:\mathrm{l}\mathrm{n}\left(\frac{P\left(Y\ge\:k|\theta\:,G\right)}{P\left(Y<k|\theta\:,G\right)}\right)={\beta\:0}_{k}+\beta\:1*\theta\:+\beta\:2*G$$

Model 3: $$\eqalign{ & \>{\rm{ln}}\left({{{P\left({Y \ge \>k|\theta \>,G} \right)} \over {P\left({Y < k|\theta \>,G} \right)}}} \right) = \beta \>{0_k} + \beta \>1*\theta \> \cr & + \beta \>2*G + \beta \>3*\theta \>*G \cr}$$

where *G* represents the nominal group variable and *θ* the construct the item is purported to measure, $\:\mathrm{l}\mathrm{n}\left(\frac{P\left(Y\ge\:k|\theta\:,G\right)}{P\left(Y<k|\theta\:,G\right)}\right)$ represents the log odds of an item response *Y* in category *k* or higher, given the level of the construct and group membership of the respondent (*k* = 1, 2 … *k*, for an ordinal item with response categories 0, 1 … *k*). *β*0, *β*1, *β*2 and *β*3 are regression coefficients of which *β*0 (the intercept) depends on the response category *k*. A statistically significant *β*2 coefficient indicates uniform DIF, whereas a statistically significant *β*3 coefficient indicates nonuniform DIF. Because statistical significance does not necessarily imply clinical significance, effect size measures were used to identify DIF that matters. An approach often used in OLR is to evaluate the difference (Δ) in R^2^ between two models. ΔR^2^ between model 1 and model 2 can be used to judge the effect size of uniform DIF, whereas ΔR^2^ between model 2 and 3 can be used to judge the effect size of nonuniform DIF. Note, that the total effect of DIF can be judged by the ΔR^2^ between model 1 and model 3. The latter is beneficial because DIF is often mixed uniform and nonuniform [25]. 

As a measure of the construct (*θ*), the so called “matching variable”, the sum of the items of the scale can be used. However, DIF in the matching variable may obscure DIF (“hidden DIF”) in some items or produce spurious DIF (“pseudo DIF”) in others. Therefore, the matching variable needs to be “purified” (i.e., stripped of all DIF). So, an iterative approach is used to identify all items with possible DIF and to remove them from the matching variable. When the matching variable is free of DIF, the for DIF suspected items can be tested one-by-one against a matching variable that includes only DIF-free items plus the item to be tested [26]. We used the following criteria for DIF: [1] a statistically significant (*p* < 0.05) *β*2 or *β*3 coefficient, and [2] a ΔR^2^ between model 1 and model 3 ≥ 0.02 [27]. In order to avoid incorrectly dismissing DIF when in reality there was DIF (type II error), we chose a relatively low cutoff for ΔR^2^ and did not apply a correction for multiple testing, thus accepting the risk of incorrectly identifying DIF when in reality there was none (type I error).

Once all items with DIF were identified, the impact of the item-level DIF on the scale-level total score was evaluated by linear regression with the scale’s total score as the dependent (outcome) variable and the sum of the DIF-free items, the group membership, and their interaction as the independent (predictor) variables. Predicted scores were then plotted as a function of the DIF-free score and group. The impact of DIF on the scale level was expressed as the difference between the predicted score lines between the groups.

### Software

We used IBM SPSS Statistics for Windows v22 (IBM Corp., Armonk, NY) to prepare and organize the data. We used the “lavaan” package, version 0.6–12 for the dimensionality analysis, [28] and the “MASS” package, version 7.3–57 for the DIF analysis, [29] both within the software program R version 4.2.1.[30].

## Results

### Translation and cross-cultural adaptation

Out of the 100 potential interview participants, a total of 27 gave permission to be interviewed. Five of them were not able to participate within the timeframe of the study and two fell ill before the interview could take place. Eventually, we interviewed 20 patients.

The group of interviewed patients consisted of eleven women and nine men. At the time of the interview, the mean age of the patients was 84 years (range 72–93 years). The average duration of their vestibular symptoms was 9.6 years (median 6.5 years). The average time it took them to complete the questionnaire was 4 min and 15 s (range 2.4–8.3 min).

The VSS-SF items were generally well-interpreted by the patients. Only minor issues were mentioned. The introductory text was often skipped as patients tended to immediately start filling out the questionnaire. The minor issues were discussed by our expert team and no changes to the individual questions were deemed necessary. To make sure the introduction would be read, we decided to present it on a separate page, apart from the questions (see appendix B for the Dutch questionnaire).

### Psychometric validation

#### Patients

The characteristics of the British and the Dutch patients are described in Table 1. Gender and age composition did not significantly differ. However, compared to the British patients, the Dutch patients had less severe symptoms that had been present for a shorter time period.

#### Dimensionality

The one-factor model did not adequately fit the Dutch data (Table 2). The bifactor models inspired by the two-factor model of Wilhelmsen et al. [8] and the three-factor model of Kondo et al. [6] also demonstrated insufficient fit. To obtain acceptably fitting bifactor models, it was necessary to allow additional specific factors and item-factor paths, one with a negative factor loading (Table 3; Fig. 1). In the final model, all items loaded on the general factor, although six items only weakly (factor loading < 0.4). The factors from Wilhelmsen et al. [8] and Kondo et al. [6] can easily be recognized in the first three specific factors. The first specific factor S1 corresponded the autonomic-anxiety factor. The second specific factor S2 corresponded to Kondo et al.’s vestibular-balance symptoms of long duration factor, whereas the third specific factor S3 corresponded to Kondo et al.’s vestibular-balance symptoms of short duration factor. Item #3 (nausea, vomiting) was shared by the first two specific factors. In addition, two small factors appeared as sub-factors of the autonomic-anxiety factor: S4 reflecting cardiopulmonary symptoms, and S5 chills and hot flushes, combined with excessive sweating. Items #1 (vertigo < 20 min) and #8 (difficulty standing or walking) appeared to be indicators of only the general factor (i.e., they did not load on any of the specific factors).

In the British data, results were comparable (Tables 2 and 3), although the bifactor model appeared somewhat more complex with a number of negative paths (Fig. 1) and two additional small specific factors. Note that the sign of the factor loadings of S3 differed between the British and Dutch data; in the Dutch data, the third factor simply reflected symptoms of short duration, whereas, in the British data, the third factor reflected symptoms of short duration *as opposed to* symptoms of long duration (but with loadings reversed).

Omega-hierarchical in the Dutch data was 0.621 (Table 4), indicating that 62.1% of the VSS-SF total score variance was explained by the general factor. Table 4 also shows omega-group for the total score (0.299) which indicates that 29.9% of the total score variance was explained by the specific factors (the rest is measurement error). Table 4 also shows how much of the variance of the sum scores of the items underlying the specific factors (S1…S7) was explained by the general factor (omega-hierarchical) or the specific group factor (omega-group). Hence, the general factor appeared stronger than the specific factors in the vertigo-balance (S2 and S3) subscales. In contrast, the specific factor appeared stronger than the general factor in the autonomic-anxiety (S1) subscale. A similar pattern was visible in the British data, although the general factor appeared to be somewhat stronger than in the Dutch data, at the expense of the specific factors.

In the Dutch data, the estimated structural coefficient was 0.433, which indicates a relative structural coefficient bias of (0.5–0.433)/0.5 = 13.4%. In the British data, the structural coefficient was 0.446, suggesting a relative bias of 10.8%. In both datasets, the relative structural coefficient bias was below 15%, suggesting that the VSS-SF was essentially unidimensional in both language versions.

#### Reliability

Omega-total coefficient was 0.920 for the Dutch questionnaire, indicating that the proportion of reliable variance in the VSS-SF total score, accounted for by the general factor and all specific factors, was 92%. For the British version, omega-total was 0.956. For comparison, Cronbach’s alpha coefficient was 0.808 for the Dutch version and 0.874 for the British version.

### Differential item functioning

When testing the items against a matching variable including all items, eight items showed significant *β*2 values suggesting possible uniform DIF and one item showed a significant *β*3 value suggesting possible nonuniform DIF. However, only three items (#3, #12 and #13) showed ΔR^2^ values ≥ 0.02. After purification of the matching variable, item #12 no longer fulfilled our DIF criteria (ΔR^2^ between model 1 and model 3 was 0.0198; Table 5). Item #3 (nausea, vomiting) was ”more severe” for Dutch patients, meaning that they were less likely to score on this item, compared to British patients, given their level of vestibular symptoms (Fig. 2, upper panel). Item #13 (unsteadiness, less than 20 min) appeared to be “less severe” for Dutch patients, meaning that Dutch patients were more likely to score this item, compared to British patients (Fig. 2, lower panel). The effect of DIF on the VSS-SF sum score is shown in Fig. 3. Because the two DIF items favored different groups, partial “DIF cancelation” occurred and an effect on the scale score appeared to be negligible.

## Discussion

### Main findings

We successfully translated and culturally adapted the VSS-SF for the Dutch population, showing that items were well understood and psychometrically sound. Importantly, our analyses go beyond routine validation. Using bifactor modeling, we demonstrated that the VSS-SF is essentially unidimensional in both Dutch and British contexts, supporting the use of a total score in clinical and research settings. DIF analyses revealed meaningful cross-cultural variation: Dutch patients reported nausea/vomiting less frequently and unsteadiness more frequently than British patients at comparable symptom levels. Although these item-level differences did not affect total scores, they highlight differences in how vestibular symptoms are perceived and communicated across healthcare contexts.

### Strengths and limitations

Strengths of the study are our strict adherence to international guidelines for translation and cross-cultural validation of questionnaires, the use of sophisticated psychometric methods, and our relatively large sample sizes. A limitation is related to the composition of our study populations. All patients were older adults with chronic vestibular symptoms. Whether the Dutch VSS-SF performs the same in younger patients and patients with acute vestibular symptoms, is an open question. In any case, older patients with chronic vestibular symptoms typically represent the target population of the VSS-SF, given its use in trials in this group [31].

### Comparison with existing literature

The factor structure revealed that, apart from a general factor, there were five specific factors underlying the Dutch VSS-SF sum scores (and seven factors underlying the British sum scores). This seems to be at variance with earlier exploratory factor analysis results [6, 8]. However, on closer examination, our first three specific factors appeared to correspond to the Kondo et al.’s factor solution [6]. Still, we needed two (Dutch) and four (British) extra specific factors to obtain acceptable fit of the factor models. Acceptable fit implies that most of the reliable variance in the item set is captured by the factors. However, the additional specific factors appeared to consist of only two or three items, hardly enough to define a factor. These weak factors may not be reproducible in other datasets. It is our experience with real data that acceptable fit is rarely obtained by specifying only substantively important and replicable item-factor relationships. Usually, it is necessary to specify minor item-factor relationships, which have arisen due to sample-specific conditions or by sheer coincidence, and which are not necessarily replicable. Compared with previous validations that relied mainly on exploratory factor analysis [6, 8], our use of bifactor modeling provided a more rigorous test of dimensionality and allowed a direct cross-national comparison between culturally distinct samples. It should be noted that the main purpose of the bifactor analysis was to partial out the general factor and assess its strength, relative to the specific factors and measurement error, in order to assess the appropriateness of using VSS-SF total scores (instead of subscale scores). In that context, the specific factors are of minor importance. Thus, despite its undeniable multidimensionality, the VSS-SF proofed to be essentially unidimensional, i.e., unidimensional enough to justify the use of total sum scores. Moreover, subtle but consistent cultural differences in item functioning suggest that linguistic equivalence alone does not fully remove cross-cultural variation in symptom reporting.

### Implications and significance

The Dutch version of the VSS-SF is a relevant addition to other available Dutch tools to assess vestibular symptoms. Another questionnaire that was previously translated into Dutch is the Dizziness Handicap Inventory (DHI) [33]. Globally, the DHI questionnaire is the most used questionnaire in vestibular medicine and can be used to measure dizziness-related impairment [33]. In daily practice, the Dutch VSS-SF can now complement the DHI. General practitioners can choose the VSS-SF when they would like to determine the *severity* (i.e., frequency) of vestibular symptoms, and they can employ the DHI when they want to quantify the *impact* of these symptoms on daily life. The VSS-SF and DHI can also be used to assess treatment effects, because cut-off points for clinically relevant changes have previously been established for both questionnaires [13, 34]. Beyond its national relevance, our findings also demonstrate that the Dutch and British VSS-SF can be meaningfully compared across countries, supporting pooled analyses in multinational trials. This shows that advanced psychometric methods such as bifactor modeling and DIF analysis can enhance cross-cultural validation by distinguishing true construct equivalence from cultural differences in symptom perception. By increasing the toolbox of both the general practitioner in clinical practice and the researcher who studies vestibular symptoms, we may increase our understanding of dizziness and ultimately improve management of symptoms.

## Conclusions

The Dutch VSS-SF is a reliable and valid tool for assessing vestibular symptoms, equivalent to the British version. Our study contributes novel evidence that, despite minor cross-cultural differences in the interpretation of specific symptoms, the VSS-SF functions as an essentially unidimensional measure across British and Dutch populations. This highlights both the robustness of the instrument and the added value of advanced psychometric approaches in uncovering cultural nuances in symptom reporting. Together, these findings strengthen the evidence base for using the VSS-SF as a standardized outcome measure in both national and international research.

**Table 1:** Patient characteristics

Characteristics	British (*n* = 296)	Dutch (*n* = 322)	*P*-value†
Female, n (percentage)	197 (67)	197 (61)	0.165
Age in years, mean (SD)	67.4 (10.2)	67.0 (9.5)	0.689
Time since vestibular diagnosis, n (percentage)*			< 0.001**
One to six months Six months to two years Two years to 10 years More than 10 years	25 (8)	50 (16)	
	63 (21)	94 (29)	
	126 (43)	123 (38)	
	80 (27)	52 (16)	
VSS-SF total score, mean (SD)	15.4 (10.2)	13.7 (8.6)	0.023**

† Differences between British and Dutch samples; independent-samples t tests for continuous variables; chi-square test for ordinal variable

* Data missing for two British participants and three Dutch participants

** Statistically significant, P-value < 0.05

**Table 2:** Results from the confirmatory factor analyses of the VSS-SF; scaled fit statistics

VSS-SF version/model	Chi-2	df	*P*	CFI^a^	TLI^b^	RMSEA (90%CI)^c^	SRMR^d^
**Dutch**							
One-factor model	703.8	90	0.000	0.713	0.665	0.146 (0.136, 0.156)	0.137
Bifactor model (2 specific factors)^e^	190.6	75	0.000	0.946	0.924	0.069 (0.057, 0.082)	0.066
Bifactor model (3 specific factors)^f^	199.2	75	0.000	0.942	0.919	0.072 (0.060, 0.084)	0.066
Modified bifactor model^g^	139.7	72	0.000	0.968	0.954	0.054 (0.041, 0.067)	0.058
**British**							
One-factor model	608.1	90	0.000	0.821	0.791	0.140 (0.129, 0.150)	0.121
Bifactor model cf. (2 specific factors)^e^	NA^h^	NA	NA	NA	NA	NA	NA
Bifactor model cf. (3 specific factors)^f^	261.1	75	0.000	0.936	0.910	0.092 (0.080, 0.104)	0.071
Modified bifactor model^g^	102.8	65	0.002	0.987	0.979	0.044 (0.027, 0.060)	0.039

^a^ CFI = comparative fit index (adequate fit if > 0.95)

^b^ TLI = Tucker-Lewis index (adequate fit if > 0.95)

^c^ RMSEA = root mean squared error of approximation with 90% confidence interval (adequate fit if < 0.06)

^d^ SRMR = standardized root mean squared residuals (adequate fit if < 0.08)

^e^ Specific factors in accordance with the Wilhelmsen et al. model [8]

^f^ Specific factors in accordance with the Kondo et al. model [6]

^g^ See Fig. 1

^h^ The model did not converge

**Table 3:** Standardized factor loadings of the unidimensional models and the final bifactor models

Dataset/Items		Uni^a^	Gen^b^	S1^c^	S2	S3	S4	S5	S6	S7
**Dutch**										
1	Vertigo (< 20 min)	0.403	0.468							
2	Chills or hot flushes	0.599	0.306	0.661				0.425		
3	Nausea, vomiting	0.523	0.399	0.362	0.276					
4	Vertigo (> 20 min)	0.692	0.515		0.691					
5	Heart pounding	0.527	0.236	0.624			0.324			
6	Dizziness (all day)	0.747	0.698		0.457					
7	Headache	0.563	0.510	0.374						
8	Difficulty standing or walking	0.546	0.651							
9	Shortness of breath	0.467	0.284	0.316			0.626			
10	Unsteadiness (> 20 min)	0.793	0.685		0.560					
11	Excessive sweating	0.614	0.396	0.533				0.425		
12	Feeling faint	0.557	0.497	0.389						
13	Unsteadiness (< 20 min)	0.513	0.638		-0.458	0.525				
14	Chest pain	0.518	0.250	0.455			0.603			
15	Dizziness (< 20 min)	0.559	0.534			0.525				
**British**										
1	Vertigo (< 20 min)	0.526	0.591				-0.205			
2	Chills or hot flushes	0.609	0.409	0.557				0.447		
3	Nausea, vomiting	0.651	0.485	0.411	0.456					
4	Vertigo (> 20 min)	0.754	0.635		0.521	0.526				
5	Heart pounding	0.655	0.500	0.589						
6	Dizziness (all day)	0.650	0.554		0.544				0.470	
7	Headache	0.598	0.462	0.483					0.470	
8	Difficulty standing or walking	0.671	0.743							
9	Shortness of breath	0.485	0.473	0.292	-0.364		0.698			
10	Unsteadiness (> 20 min)	0.816	0.784		0.324	0.214				0.433
11	Excessive sweating	0.611	0.412	0.541		0.264		0.447		
12	Feeling faint	0.679	0.600	0.425						
13	Unsteadiness (< 20 min)	0.730	0.771			-0.316				
14	Chest pain	0.442	0.253	0.476		-0.232	0.470			
15	Dizziness (< 20 min)	0.671	0.724			-0.366				-0.433

^a^ Uni = factor of the unidimensional model

^b^ Gen = general factor of the bifactor model

^c^ S1 … S7 = specific factors of the bifactor model

**Table 4:** Omega coefficients to evaluate the strength of the general and specific factors

Dataset/Statistic	Gen^a^	S1^b^	S2	S3	S4	S5	S6	S7
**Dutch**								
Omega-hierarchical	0.621	0.321	0.535	0.454	0.139	0.262		
Omega-group	0.299	0.534	0.370	0.365	0.568	0.384		
**British**								
Omega-hierarchical	0.691	0.433	0.606	0.722	0.367	0.327	0.425	0.705
Omega-group	0.265	0.477	0.343	0.207	0.398	0.387	0.365	0.233

^a^ Gen = general factor of the bifactor model

^b^ S1 … S7 = specific factors of the bifactor model

**Table 5:** Results of the DIF analysis

Item	$\:{\varDelta\:\mathbf{R}}_{\mathrm{1,2}}^{2}$	$\:{\varDelta\:\mathbf{R}}_{\mathrm{1,3}}^{2}$	*P*(β2)	*P*(β3)
1	0.000	0.002	0.679	0.198
2	0.017	0.019	0.000	0.206
3	0.043	0.043	0.000	0.488
4	0.009	0.009	0.010	0.948
5	0.009	0.014	0.007	0.046
6	0.002	0.014	0.208	0.001
7	0.002	0.003	0.125	0.595
8	0.011	0.012	0.002	0.319
9	0.000	0.001	0.788	0.441
10	0.001	0.001	0.503	0.490
11	0.007	0.007	0.019	0.926
12	0.019	0.020	0.000	0.566
13	0.021	0.022	0.000	0.316
14	0.003	0.007	0.214	0.108
15	0.000	0.000	0.621	0.959

DIF= differential item functioning; Items that show DIF are highlighted

$\:{\varDelta\:\mathrm{R}}_{\mathrm{1,2}}^{2}$=$\:{\varDelta\:\mathrm{R}}^{2}$difference between model 1 and model 2

$\:{\varDelta\:\mathrm{R}}_{\mathrm{1,3}}^{2}$=$\:{\varDelta\:\mathrm{R}}^{2}$difference between model 1 and model 3

P(*β*2) = P-value of *β*2 coefficient (for group)

P(*β*3) = P-value of *β*3 coefficient (for group*matching variable)

## Supplementary Information

Below is the link to the electronic supplementary material.

Supplementary Material 1

Supplementary Material 2

## Data Availability

The datasets used and/or analyzed during the current study are available from the corresponding author on reasonable request.
