# Supplementary material for: Cross-cultural validation of the Dutch Vertigo Symptom Scale – Short Form: dimensionality, reliability and cultural differences in symptom reporting
Source: J Patient Rep Outcomes. 2026 Jun 10;10:146. doi: 10.1186/s41687-026-01109-x (PMC13481963; doi:10.1186/s41687-026-01109-x)
Supplement: Supplementary file 1 [file 41687_2026_1109_MOESM1_ESM.pdf]

Vertigo symptom scale – short form (Nederlandse versie)

We willen graag weten welke, aan duizeligheid gerelateerde, klachten u recent heeft gehad. Kies alstublieft het meest geschikte hokje om aan te geven hoe vaak u in de afgelopen maand deze klachten heeft ervaren. Vul dit hokje in met een duidelijke “X”.

Hoe vaak heeft u in de afgelopen maand last gehad van één of meer van de volgende symptomen?

		**Nooit**	**Een enkele keer**	**Verschil-lende keren**	**Vaak (elke week)**	**Meestal (bijna elke dag)**
1	Een gevoel dat u of de wereld om u heen ronddraait of beweegt gedurende korter dan 20 minuten.					
2	Plots gevoel van kou of warmte.					
3	Misselijkheid, overgeven.					
4	Een gevoel dat u of de wereld om u heen ronddraait of beweegt gedurende langer dan 20 minuten.					
5	Hartbonzen of hartkloppingen.					
6	Duizeligheid, wazigheid of desoriëntatie die de hele dag aanhoudt.					
7	Hoofdpijn of een gevoel van druk in het hoofd.					
8	Niet goed kunnen staan of lopen zonder ondersteuning, wankelen of afwijken naar één kant.					
9	Moeite met ademhalen, kortademigheid.					
10	Onstabiel gevoel, bijna uit evenwicht raken, gedurende langer dan 20 minuten.					
11	Overmatig zweten.					
12	Het gevoel alsof u elk moment flauw kunt vallen.					
13	Onstabiel gevoel, bijna uit evenwicht raken, gedurende korter dan 20 minuten.					
14	Pijn in de hartstreek of borstkas.					
15	Duizeligheid, wazigheid of desoriëntatie die korter dan 20 minuten aanhoudt.					
