# Supplementary material for: Cross-cultural validation of the Dutch Vertigo Symptom Scale – Short Form: dimensionality, reliability and cultural differences in symptom reporting
Source: J Patient Rep Outcomes. 2026 Jun 10;10:146. doi: 10.1186/s41687-026-01109-x (PMC13481963; doi:10.1186/s41687-026-01109-x)
Supplement: Supplementary file 2 [file 41687_2026_1109_MOESM2_ESM.pdf]

Vertigo symptom scale – short form (original version)

We would like to know what dizziness-related symptoms you have had recently. Please select the appropriate box to indicate how many times you have experienced each of the symptoms listed below during the past month. Please fill out the box with a clear “X”.

How often in the past <u>month</u> have you experienced one or more of the following symptoms?

		**Never**	**A few times**	**Several times**	**Quite often (every week)**	**Very often (most days)**
1	A feeling that either you, or things around you, are spinning or moving, lasting less than 20 minutes					
2	Hot or cold spells					
3	Nausea (feeling sick), vomiting					
4	A feeling that either you, or things around you, are spinning or moving, lasting more than 20 minutes					
5	Heart pounding or fluttering					
6	A feeling of being dizzy, disoriented or "swimmy", lasting all day					
7	Headache, or feeling of pressure in the head					
8	Unable to stand or walk properly without support, veering or staggering to one side					
9	Difficulty breathing, been short of breath					
10	Feeling unsteady, about to loose balance, lasting more than 20 minutes					
11	Excessive sweating					
12	Feeling faint, about to black out					
13	Feeling unsteady, about to loose balance, lasting less than 20 minutes					
14	Pains in the heart or chest region					
15	A feeling of being dizzy, disoriented or "swimmy", lasting less than 20 minutes					
